# The development of a consensus-based nutritional pathway for infants with CHD before surgery using a modified Delphi process

**DOI:** 10.1017/S1047951118000549

**Published:** 2018-04-29

**Authors:** Luise V. Marino, Mark J. Johnson, Nigel J. Hall, Natalie J. Davies, Catherine S. Kidd, M. Lowri Daniels, Julia E. Robinson, Trevor Richens, Tara Bharucha, Anne-Sophie E. Darlington

**Affiliations:** 1 Department of Dietetics/SLT, University Hospital Southampton NHS Foundation Trust, Southampton, UK; 2 NIHR Biomedical Research Centre Southampton, University Hospital Southampton NHS Foundation Trust and University of Southampton, Southampton, UK; 3 Department of Neonatal Medicine, University Hospital Southampton NHS Foundation Trust and University of Southampton, Southampton, UK; 4 Department of Surgery, University Hospital Southampton NHS Foundation Trust and University of Southampton, Southampton, UK; 5 ^5^Faculty of Medicine, University of Southampton, Southampton, UK; 6 Department of Paediatric Cardiology, University Hospital Southampton NHS Foundation Trust and University of Southampton, Southampton, UK; 7 Faculty of Health Sciences, University of Southampton, Southampton, UK

**Keywords:** CHD, infants, nutrition, growth, Delphi

## Abstract

**Introduction:**

Despite improvements in the medical and surgical management of infants with CHD, growth failure before surgery in many infants continues to be a significant concern. A nutritional pathway was developed, the aim of which was to provide a structured approach to nutritional care for infants with CHD awaiting surgery.

**Materials and methods:**

The modified Delphi process was development of a nutritional pathway; initial stakeholder meeting to finalise draft guidelines and develop questions; round 1 anonymous online survey; round 2 online survey; regional cardiac conference and pathway revision; and final expert meeting and pathway finalisation.

**Results:**

Paediatric Dietitians from all 11 of the paediatric cardiology surgical centres in the United Kingdom contributed to the guideline development. In all, 33% of participants had 9 or more years of experience working with infants with CHD. By the end of rounds 1 and 2, 76 and 96% of participants, respectively, were in agreement with the statements. Three statements where consensus was not achieved by the end of round 2 were discussed and agreed at the final expert group meeting.

**Conclusions:**

Nutrition guidelines were developed for infants with CHD awaiting surgery, using a modified Delphi process, incorporating the best available evidence and expert opinion with regard to nutritional support in this group.

## Background

CHD represents one-third of all major congenital anomalies, with a reported prevalence of 9 per 1000 live births [95% CI: 8.1-9.3]. During the past 50 years, there have been significant improvements in the medical and surgical management of CHD, with more children now reaching adulthood.^1^ With improved survival comes an increasing burden of morbidity. In particular, growth failure during the first 2 years of life is considered to be a significant concern in infants with CHD.^2^ ^–^ ^6^ World Health Organisation definitions of persistent malnutrition in children include “stunting”, with a height for age⩽−2 z scores, and “underweight”, with a weight for age⩽−2 z score.^7^ Persistent malnutrition in childhood is important as it has been linked to shorter adult height, increased all-cause mortality,^8^ as well as poorer neurodevelopmental outcomes among young children with CHD.^9^

Stunting and becoming underweight are both dynamic processes of persistent malnutrition and are indicative of insufficient macronutrients and micronutrients to promote adequate growth.^10^ The prevalence of persistent malnutrition at the time of CHD surgery is reportedly 30%,^6^ ^,^ ^11^ leading to poorer postoperative resilience and clinical outcomes including increased risk of cardiac arrest and infection,^12^ prolonged ICU stay,^13^ and length of hospital stay.^6^ ^,^ ^14^ In addition, infants with CHD who are underweight for age at the time of surgery also experience significant morbidity,^15^ ^,^ ^16^ and those who are slow to gain weight postoperatively have increased mortality at 3 months of age.^17^

Growth failure among infants is not just restricted to those with complex CHD lesions; infants with ventricular septal defects are often severely underweight at the time to surgery. As such, facilitating better growth before surgery has been seen as key to improving short- and longer-term outcomes,^18^ particularly as rapid catch-up growth after infancy is associated with negative metabolic sequela. By 2 years of age, many young children with CHD will have undergone surgery for their condition. However, a high-risk growth pattern has been defined as growth failure during the first 2 years of life with subsequent rapid catch-up growth between the ages of 2 and 7 years and 8 and 15 years.^19^ The current consequence of these growth patterns with respect to CHD is unknown, but it is speculated that increased adiposity in adults with CHD is associated with an increased risk of metabolic and cardiovascular disease later in life.^20^ ^–^ ^22^ As a result, sustaining inherited growth patterns in infants with CHD before surgery, thereby avoiding rapid late catch-up postoperative growth, is fundamental to reducing long-term co-morbid complications.^19^

A number of quality improvement initiatives such as home monitoring programmes that aim to facilitate better growth during the months before surgery, particularly in those infants requiring a staged surgical approach, for example, univentricular physiology, have been implemented.^18^ ^,^ ^23^ ^–^ ^25^ However, even within these well-established programmes, nutritional pathways describing principles to optimise nutritional support are not available.^26^ ^,^ ^27^ Variations in nutrition practice^15^ ^,^ ^26^ ^,^ ^27^ may contribute to sub-optimal growth in the period leading up to surgery.^27^ Although there is a body of evidence around nutritional needs of infants with CHD, as well as a number of published algorithms with regard to nutritional support in the immediate postoperative period,^28^ ^–^ ^31^ to our knowledge none exist to support of infants in the months leading up to cardiac surgery. Variation in care across different units may contribute to differences in surgical outcomes, and there is a move towards standardising care aligned to defined standards to reduce the risks associated with variations in practice. In addition, lack of consensus regarding nutritional support in infants with CHD causes parental distress owing to conflicting messages.^4^ ^,^ ^32^ To address this gap, we aimed to develop a consensus-based nutritional pathway providing a structured approach for the nutritional care of infants with CHD awaiting surgical palliation or repair.

## Methods

**Figure 1:**
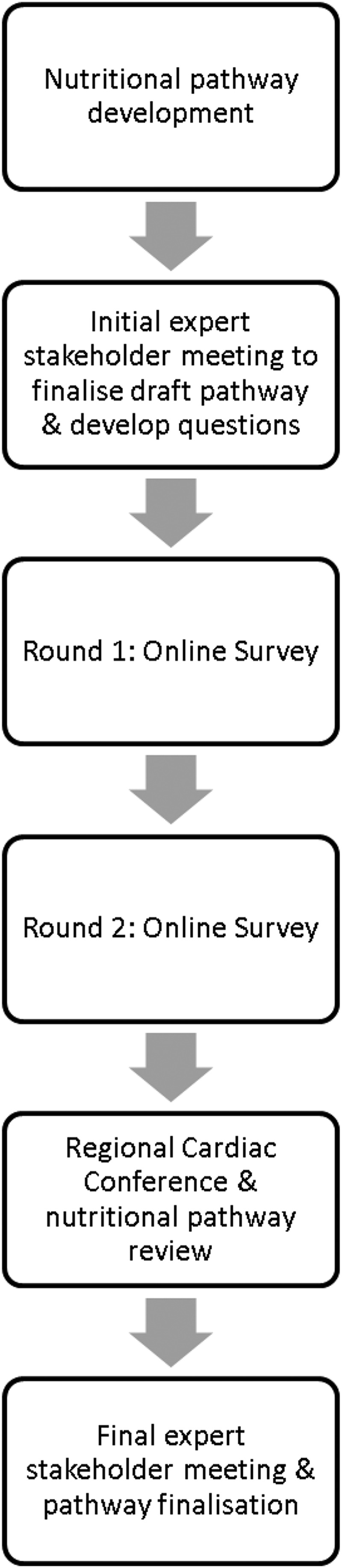
Process followed during modified Delphi consensus.

To develop the nutritional pathway to be used by paediatric dietitians, and other healthcare professionals, in the support of infants with CHD before surgery we used the modified Delphi consensus method described by Keller et al^33^ (Fig 1). Initially, we developed a set of principles to guide development of the nutritional pathway to help ensure that key objectives were met. Existing nutritional pathways or guidelines that had used a systematic evidence-based approach to nutritional support of infants during the perioperative period^28^ ^,^ ^29^ were modified following a focused literature search. The contributing literature is summarised in Supplementary material 1. The draft pathway was based on principles outlined in the Word Health Organizations Integrated Management of Childhood Illness. The aim was to provide a simple nutritional pathway based on a traffic light system of green (no concern), amber (some concern), and red (significant concern).^34^ The draft pathway was reviewed and refined by a small working group of investigators (L.V.M., N.J.D., C.S.K., M.L.D., J.E.R., M.J.J., and T.B.) before being presented at the first expert stakeholder meeting.

### Step 1: First expert stakeholder meeting of British Paediatric Dietetic Paediatric Cardiology Interest Group

An expert stakeholder meeting was held with members of the British Dietetic Association (BDA) Paediatric Cardiology Interest Group who are also paediatric dietitians from Tertiary Surgical Cardiac Centres. The purpose of the meeting was to review and discuss the initial draft pathway and the planned consensus process in addition to gaining agreement behind the nutritional principles that had been incorporated from the available evidence.^26^ ^,^ ^28^ ^–^ ^30^

### Step 2: Development of Delphi statements and open-ended questions

After the first expert stakeholder meeting, changes were made to the draft pathway after which statements to be used in the two rounds of an online survey were developed. The survey contained 31 questions split into five sections, representing the layout of the nutritional pathway. For each question, participants were asked to indicate their level of agreement with a statement and responded using a 10-point scale, with 1 indicating strongly disagree to 10 indicating strongly agree, which included a neutral option. At the end of each section, participants were provided with the opportunity to include additional comments, within an open-ended text box (Supplementary material 2). Participant responses accounted for only one rating per question.

### Stage 3: Two rounds of the online Delphi survey

The survey was created and distributed through a proprietary online platform hosted by the University of Southampton (iSurvey: https://www.isurvey.soton.ac.uk/). Members of the BDA Paediatric Cardiology Interest Group were invited to complete the first round of survey and sent a reminder after 3 weeks. Responses to each question were grouped into “disagreement” 1–4 and “agreement” 7–10. For analysis, consensus was defined as ⩾80% responses for each question as either “disagreement” or “agreement”.^35^ In round 2 of the survey, the questions remained unchanged, and participants were provided their own score from round 1 along with the cumulative scores from the rest of the group. They were invited to consider their score in comparison with the group score and offered the opportunity to modify their own score in light of this should they wish. It was made clear that even with the additional information provided participants did not have to change their opinion. Participants were given 4 weeks to complete the second round, as it was during high peak summer holiday season. Participants were informed that changes would be made to the draft pathway on the basis of consensus achieved after 2 rounds. Participants provided written consent as part of the Delphi survey.

### Step 4: Regional conference: nutrition support in infants with CHD

As Paediatric Cardiac networks cover wide geographic areas, nutritional support is provided by paediatric dietitians working in a District General Hospital, as well as specialist centres. It was felt important to ensure that dietitians working in these hospitals agreed with the principles and content of the nutritional pathway in advance of the final expert meeting. Two months before the final expert meeting, clinical staff from NHS District General Hospitals (South Central Region, UK) were invited to attend a regional cardiac nutrition conference to discuss the modified pathway and achieve consensus with the nutrition principles outlined in the pathway across a wider group. Participants registered for the meeting were sent a copy of the modified nutritional pathway in advance. The morning session of the meeting was dedicated to presentations on nutritional support of infants with CHD, and set the scene for the development of the nutrition pathway. Participants registered for the meeting were sent a copy of the nutritional pathway in advance to be used as part of the afternoon facilitated discussion by M.J.J., who led the group through a point-by-point group discussion of the format and contents of the draft pathway. Paper copies were also printed for the day itself.

### Stage 5: Second and final expert stakeholder meeting

A final face-to-face expert stakeholder meeting of BDA Paediatric Cardiology Interest Group was held whereby L.V.M. led the group through a point-by-point group discussion of the format and contents of the draft pathway, including areas of contention, with the aim of confirming the final version of the nutritional pathway for infants with CHD before surgery.

## Results

### Step 1: First expert stakeholder meeting of BDA Paediatric Cardiology Interest Group

In total, 10 expert dietitians from the BDA Paediatric Cardiology Interest Group and one physician attended the first stakeholder meeting (Table 1). During the point-by-point discussion, iterative changes were made to CHD conditions, with transposition of the great arteries move to higher nutritional risk, in addition to protein requirements for those with lower nutritional risk. By the conclusion of the meeting, all present agreed on the process of consensus in addition to the draft nutritional pathway.

**Table 1:** Characteristics of expert stakeholders and regional meeting of healthcare professionals.

Profession	Initial expert stakeholder meeting (n=10)	1st round Delphi survey (n=20)	2nd round Delphi survey (n=15)	Final stakeholder (n=16)	Regional meeting (n=42)
Physician	1	3	1	0	5
Dietitian	10	17	13	16	32
Nurses	0	0	0	0	4
Speech and language therapist	0	1	1	0	1
Organisations represented	1, 2, 3, 4, 5, 6, 10, 11	1, 2, 3, 4, 5, 6, 7, 8, 9, 10, 11, 12, 13, 14	1, 2, 3, 4, 5, 6, 8, 9, 10, 11, 12, 13, 14	1, 2, 3, 4, 5, 6, 8, 10, 11, 12, 13, 14	11, 12, 14–31

Specialist Cardiac Level 3 Centres: 1=Alder Hey Children’s Hospital NHS Foundation Trust; 2=Glasgow Children’s Hospital NHS Trust; 3=University Hospitals of Leicester NHS Trust; 4=Royal Brompton & Harefield NHS Foundation Trust; 5=Evalina Children’s Hospital NHS Foundation Trust; 6=Great Ormond Street Hospital for Children NHS Foundation Trust; 7=Newcastle Hospitals NHS Foundation Trust; 8=University Hospitals Bristol NHS Foundation Trust; 9=Leeds Teaching Hospitals NHS Trust; 10=Birmingham Children’s Hospital NHS Foundation Trust; 11=University Hospital Southampton NHS Foundation Trust, Others: 12=Our Lady’s Hospital, Dublin; 13=HCA Hospital, London; 14=Yeoville NHS District General Hospital; 15=St Peter’s NHS Hospital, Chichester; 16=Queen Alexandre NHS Foundation Hospital, Portsmouth; 17=Dorchester NHS Hospital; 18=Frimley NHS Hospital; 19=St. Mary’s NHS Hospital, Isle of Wight; 20=Kings College Hospital NHS Foundation Trust, London; 21=John Radcliffe NHS Foundation Trust, Oxford; 22=Stoke Mandeville NHS Foundation Trust Hospital; 23=Milton Keynes University Foundation Trust, Milton Keynes; 24=Reading NHS Foundation Hospital, Reading; 25=Worthing NHS Hospital, Worthing; 26=Bart Health NHS Foundation Trust, London; 27=Kings College Hospital NHS Foundation Trust, London; 28=Cardiff University Hospital Cardiff, Wales; 29=Barking NHS Hospital, London; 30=Royal Surrey County Hospital, Guildford; 31=Bromley Health Care, Bromley

### Step 2: Development of Delphi statements and open-ended questions

On the basis of the initial draft guidelines, survey questions were created and the survey distributed to registered participants. The survey is detailed in Supplementary material 2.

The results for each question were exported to an excel file (csv) for review and analysis. Qualitative content was used for the comments, with minimal interpretation. All open-ended comments from rounds one and two were presented at the final expert stakeholder meeting to ensure that all opinions were accounted for.

### Stage 3: Two-round online Delphi survey

An initial e-mail explaining the process and purpose of the Delphi consensus was sent to all members of the BDA Paediatric Cardiology Interest Group, who were asked to forward the e-mail onto other colleagues working in Paediatric Cardiology within their organisation who may be interested in participating. In all, 35 expert healthcare professionals expressed interest in completing the online survey. After their expression of interest, a 2nd e-mail was sent with a URL link to access the survey in addition to instructions for completion. Of the 35 registered, 20 completed round 1 (57%), including two clinicians and 18 dietitians from the BDA Paediatric Cardiology Interest Group. Of the 20 healthcare professionals who completed the first round, 15 (75%) completed round 2 (Table 2). Given the small number of specialist paediatric dietitians working in Tertiary Cardiac Surgical centres UK (n=18), the response rate for the survey was considered good as there was representation from each of the Tertiary Surgical Cardiac Centres. Of paediatric dietetic participants, one-third had more than 9 years of experience working with infants with CHD.

**Table 2:** Principles supporting the development of the nutrition pathway for infants with CHD before surgery.

The nutritional process within the guidelines will: ∙ Provide a structured process by which nutritional risk in the infant with CHD can be identified, with the aim of improving growth in infants before surgery ∙ Focuses on nutritional support in infants with CDH before surgery ∙ Will be feasible and practical to use in a variety of healthcare settings in the United Kingdom ∙ Will be clinically credible ∙ Will be based on the best available evidence/practice where it exists ∙ Uses a broad set of strategies and guiding principles, which can meet the needs of the majority of infants with CHD before surgery ∙ Identifies a clear process of assessment and review for individual infants requiring input from a paediatric dietitian/speech and language therapist including time frames with regard to type of nutritional support and frequency of review ∙ Provides risk stratification based on a traffic light principle, identifying increasing levels of intervention, support, and monitoring for higher-risk patients ∙ Provides nutrition care plans, which align with individual goals for growth and incorporates the wishes of the family regarding feeding choice, ensuring the promotion and protection of breast-feeding ∙ Promotes the use of appropriately energy–nutrient-dense feed/food where applicable in conjunction with breast milk decreasing the potential for growth faltering ∙ Provides a nutrition process with role and responsibilities within this ∙ Is sufficiently specific to be able to be evaluated through a quality improvement framework including audit

After the first round, consensus was achieved regarding 76% of statements, and after round 2 this had increased to 96% (Table 3). Consensus had not been achieved regarding just three statements after round 2, namely infants with transposition of the great arteries have a high nutritional risk; an infant who does not vomit has a low nutritional risk; and infants will no longer require nutritional support 12 weeks after definitive surgery. These three statements were discussed at length during the final stakeholder meeting.

**Table 3:** Number and percentage of participant’s agreement with each statement between survey round 1 and 2.

	1st round		2nd round	
Statements used within the Delphi survey	n	%	n	%
The nutritional needs of infants with CHD will depend on the type	15	75	12	80
It is important to develop some nutrition guidelines for infant	17	85	15	100
Patent ductus arteriosus (if early surgery)	19	95	15	100
Atrial septal defect	14	70	12	80
Cor triatriatum	14	70	14	93
Total anomalous pulmonary venous drainage	16	80	14	93
Pulmonary stenosis	13	65	14	93
Coarctation of aorta	16	80	14	93
Pulmonary atresia	14	70	12	80
Tetralogy of Fallot	15	75	12	80
Atrial septal defect (severe lesion)	16	80	12	80
Transposition of great arteries	9	45	2	13
Ventricular septal defect (moderate to large)	20	100	15	100
Arterioventricular septal defect	20	100	15	100
Hypoplastic left heart syndrome	19	95	15	100
Truncus arteriosus	20	100	15	100
Aorto pulmonary window	18	90	14	93
Patent ductus arteriosus (large/delayed surgery)	17	85	15	100
Tricuspid atresia	18	90	15	100
Ebstein anomaly	18	90	15	100
Double-outlet right ventricle	16	80	15	100
Partial anomalous pulmonary venous drainage	17	85	15	100
T21/18/13	15	75	14	93
VACTERL/CHARGE	20	100	15	100
Gastrointestinal atresia	20	100	15	100
Di-George syndrome	20	100	14	93
Congenital chylothorax	20	100	15	100
Regular assessment of growth in an infant with CHD identifies	20	100	15	100
Gaining an adequate amount of weight>10 g/kg/day	20	100	13	87
Weight not more than 2 centiles below birth centile after 3 week	15	75	12	80
Following a growth curve	14	70	15	100
Not gaining adequate amounts of weight	18	90	13	87
Sustained weight drop of 2 centiles or more from birth after 3 weeks	19	95	15	100
Flattening of growth curve	20	100	15	100
Growth curve dropping downwards or losing weight	20	100	15	100
To prevent oral aversion a review by a speech and language therapist (SLT)	20	100	14	93
Shows signs of distress during or after a feed	17	85	15	100
Breathing sounds are noisy/ wet during/after a feed	17	85	15	100
Coughing, gagging, or choking episodes	20	100	15	100
Losing fluid from the mouth or fluid/food remaining in the mouth	20	100	14	93
Changes in breathing/saturation levels during a feed	18	90	15	100
An infant changes colour during or after a feed	18	90	15	100
Regression of oral feeding skills or oro-motor difficulties	18	90	15	100
Difficulty in moving from enteral feeds to oral intake	20	100	15	100
Breath-holding during a feed	19	95	14	93
Not vomit	17	85	7	53
Drink 150 ml/kg or above	7	35	14	93
Keen to drink	13	65	13	87
Finishes expected amount of infant feed	17	85	13	87
Breastfeeds for expected duration	17	85	14	93
Vomit with most feeds	14	70	14	93
Be fluid-restricted or drink <120 ml/kg	15	75	13	87
Only drinks a portion of the feed offered	19	95	12	80
Require a nasogastric tube	18	90	14	93
Growing well	17	85	15	100
Be keen to drink	20	100	15	100
A CHD lesion with a lower nutritional risk	20	100	15	100
Will require between 90 and 100 kcal/kg	17	85	14	93
Require 1.5 g/kg protein (e.g. 2 g protein per 150 ml)	16	80	14	93
Should have breast milk or standard infant formula	15	75	15	100
Weaning foods from 17 to 26 weeks age	20	100	14	93
Should be reviewed by local team	19	95	13	87
Not growing well	17	85	14	93
Do not always finish feeds offered	19	95	14	93
CHD lesion with a higher nutritional risk	17	85	14	93
Shows signs of distress during feeds	17	85	14	93
Fluid intake <120 ml/kg	16	80	13	87
Will require between 110 and 120 kcal/kg	18	90	13	87
Will require 2.5 g/kg protein	19	95	13	87
Should have breast milk/infant formula and 30–50% energy/nutrient	17	85	13	87
1 tsp nut butter in weaning foods from 17 to 26 weeks of age	17	85	12	80
Dietetic/growth review every 2 weeks	12	60	14	93
Losing weight/not growing well	17	85	15	100
CHD lesion with higher nutrition risk	19	95	15	100
Takes a long time to feed or tires easily	19	95	15	100
Has difficulty feeding	20	100	15	100
Fluid-restricted <100 ml/kg	20	100	15	100
Will require 120–150 kcal/kg	18	90	14	93
Will require up to 4 g/kg protein	20	100	13	87
Breast milk/infant formula and 50–80% energy/nutrient-dense feed	18	90	13	87
1–2 tsp nut butter in weaning foods from 17 to 26 weeks	18	90	12	80
Dietetic/growth review every week	14	70	14	93
They have achieved catch-up growth to 1 centile below birth weight	18	90	14	93
They are 12 weeks post definitive surgery	10	50	8	52

Agreement scores 7–10; disagreement scores 1–4

### Step 4: Regional conference: nutrition support in infants with CHD

**Figure 2:**
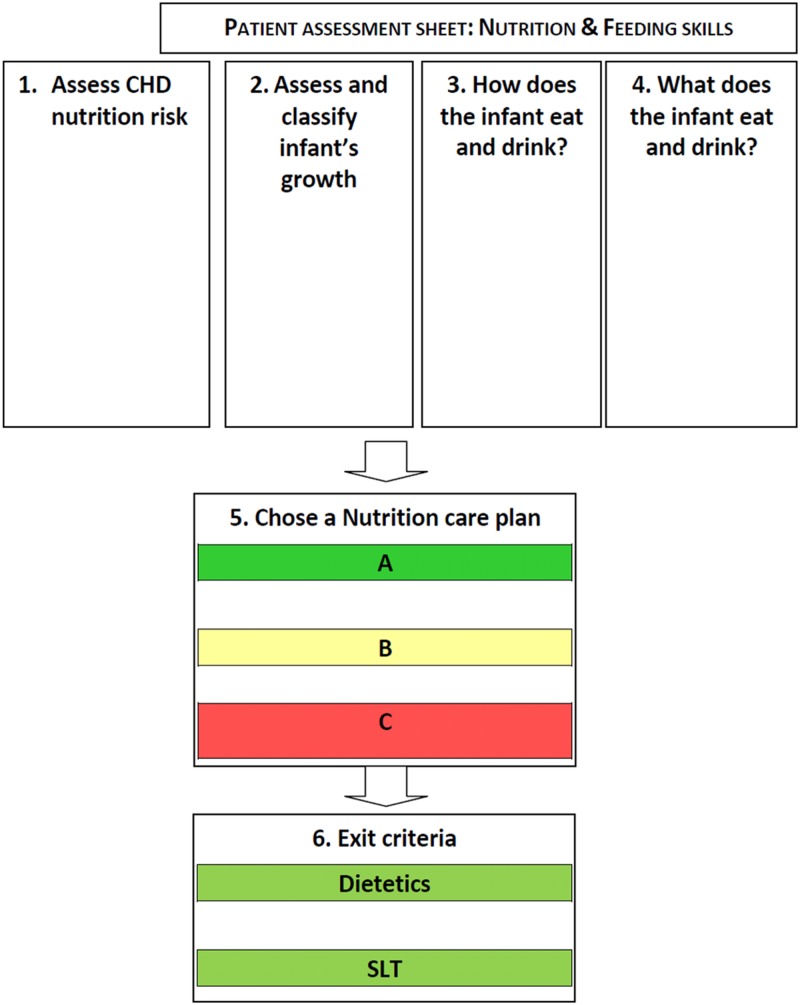
Nutritional pathway for infants with CHD before surgery. Nutrition Care Plan A, B, and C describe a package of nutritional care, in addition to exit criteria for dietetic and speech and language therapist (SLT) support (full nutritional pathway available in Supplementary material 3).

**Figure 3:**
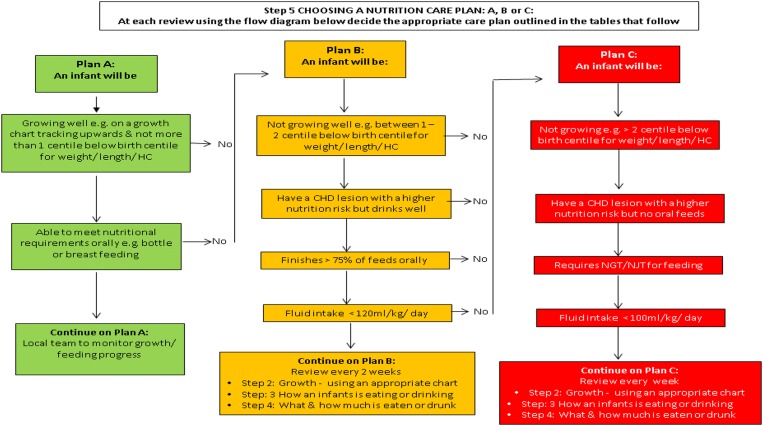
Step 5: Choosing a nutrition care plan: A, B or C (full nutritional pathway available in Supplementary material 3).

In total, 42 participants took part in the Regional Conference: *Nutrition support in infants with CHD* including five clinicians, 32 paediatric dietitians, four nurses, and one speech and language therapist, working within the Southampton-Oxford Cardiology network. The afternoon session was dedicated to the nutritional pathway, whereupon the same moderator (M.J.J.) as for the first expert stakeholder meeting led those in attendance through a point-by-point discussion of the pathway, which provided the opportunity to make further iterative changes (Fig 2). Discussion focused on ensuring that the pathway contained guidance that could be implemented in the majority of settings. The meeting facilitator (L.V.M.) recorded minutes and used this to produce a final version of the pathway. The conference participants agreed with all components of the nutrition pathway, although the group recommended that the format of the screening questions outlined in Step 5 of the pathway – “Choosing a Nutrition Care Plan A, B and C” – be changed to an algorithm (Fig 3). Subsequent to the meeting, the investigators (L.V.M., C.S.K., and M.J.J.) developed a simple algorithm for this step (Fig 3), which was presented at the final stakeholder meeting.

### Stage 5: Second and final expert stakeholder meeting

The finalised nutritional pathway was presented at the final stakeholder meeting of the BDA Paediatric Cardiology Interest Group, attended by specialist dietetic representation from all but two of the Level 3 Cardiac centres. Dietitians from those two centres had participated in the online survey. The moderator (L.V.M.) led those in the meeting through a point-by-point discussion.

The three statements on which consensus had not been reached during the Delphi process were discussed, amended, and subsequently consensus was reached to permit inclusion in the final pathway: infants with transposition of the great arteries have a high nutritional risk; an infant who does not vomit has a low nutritional risk; and infants will no longer require nutritional support 12 weeks after definitive surgery.

The format change – that is, the use of an algorithm in place of a table for Step 5 “Choosing a Nutrition Care Plan A, B and C” – was discussed during the meeting. As only the format and not the information within had changed, the group agreed on the layout change. All participants at the meeting agreed on the content and format of the finalised pathway. The final pathway presented in Supplementary material 3 has since been endorsed by the British Dietetic Association.

## Discussion

The best available evidence from the literature relating to nutritional support of infants with CHD^3^ ^,^ ^4^ ^,^ ^14^ ^,^ ^27^ ^–^ ^31^ ^,^ ^36^ ^–^ ^69^ was used to develop a nutritional pathway for infants with CHD. This was presented at an initial expert meeting involving the BDA Paediatric Cardiology Interest Group, taken through two rounds of an anonymous Delphi survey, discussed at a regional nutrition conference and finalised at a final expert BDA Paediatric Cardiology Interest Group meeting. Iterative changes were made throughout the process. At the end of this process, consensus around the nutrition principles within a nutritional pathway for infants with CHD awaiting surgery was achieved. This modified Delphi consensus process was inclusive of paediatric dietetic experts working in Tertiary Surgical Cardiac centres, as well as those working in District General Hospitals. Of those who registered to complete the Delphi survey, 17 dietitians completed the first round, of whom 80% went on to complete the second round, demonstrating a good level of engagement with the principles of the nutritional pathway. The timing of the survey – for example summer holidays – may have had an impact on participation. Consensus at the end of round 2 of the Delphi survey was achieved in all but three minor areas relating to reclassifying the nutritional risk of transposition of the great arteries, vomiting in infants, and the duration of follow-up post surgical repair. Importantly, the processes used in this project, particularly the regional conference, will have raised awareness and encouraged engagement in relation to the pathway, making successful implementation more likely going forward.

The overarching ambition of our wider quality improvement programme including the development of a nutritional pathway in infants with CHD before surgical repair/palliation was to reduce variation in nutrition management of infants with CHD; promote early referral to a paediatric dietitian/Speech and Language Therapist for feeding difficulties; reduce the prevalence of persistent malnutrition, as defined by WHO classifications, at the time of surgery; and improve clinical outcomes. Although there are a number of published algorithms with regard to nutrition support in the immediate postoperative intensive care period, until now none currently existed for the support of infants with CHD leading up to surgery.^28^ ^–^ ^31^ In an ideal setting, all infants with CHD at high risk of growth failure should be reviewed by a Paediatric Cardiac Dietitian weekly as part of a multidisciplinary team process. This action alone has been shown to improve growth among those with univentricular physiology.^15^ Currently, there is variable and often inadequate resource available within Paediatric Cardiac centres. Most units only have sufficient resource to provide nutritional support to inpatients, and on discharge patients are often referred to local dietetic services for ongoing nutrition support. A lack of consensus regarding optimal nutritional support for infants with CHD may contribute to the poor growth of infants awaiting surgery and have a negative impact on clinical outcomes.^6^ ^,^ ^13^ ^,^ ^16^ Therefore, improving growth before surgery is a priority. Persistent malnutrition has been widely described in infants with CHD including in cardiac centres in other countries.^9^ ^,^ ^11^ ^,^ ^14^ ^,^ ^70^ ^–^ ^73^ We aimed to ensure that the principles of nutritional care within the pathway were as generic as possible to allow local adaptation within a variety of healthcare settings both nationally and internationally.

The causality of growth failure in this population group is multifactorial and includes increased metabolic requirements, malabsorption, and sub-optimal nutrition intake.^46^ ^,^ ^73^ ^,^ ^74^ The Nutrition Care Plans A, B, and C were based on evidence suggesting that growth in children with complex CHD benefits from early intensive nutrition support,^75^ making use of energy- and nutrient-dense formulas where necessary.^3^ ^,^ ^46^ Within the literature, recommendations for nutrition support suggest that growth will be achieved with a calorie intake of 90–110 kcal/kg, ensuring an optimal protein–energy ratio of 9–12%^10^ and sufficient intake of micronutrients.^3^ ^,^ ^10^ ^,^ ^28^ ^,^ ^29^ ^,^ ^33^ Although energy expenditure in infants with CHD has not been shown to be increased,^61^ ^,^ ^68^ there is evidence that additional energy and protein is required to support catch-up growth.^10^ Achieving sufficient intake is often affected by vomiting, reflux, ability to sustain feeding for long enough before tiring, and early satiety.^73^

As most infants with CHD are followed up by local dietetic services rather than at a specialist centre, it was imperative to achieve wide stakeholder engagement and agreement to the nutrition principles within the nutritional pathway. This was achieved during a regional nutrition conference. All of the participants attending the conference agreed with the content of the nutritional pathway, but suggested a format change for Step 5 “Choosing a Nutrition Care Plan A, B or C” within the guidelines. This amendment was made before presenting the pathway at the final expert meeting. During the final stakeholder meeting, each of the points was discussed until consensus was achieved.

Some qualitative comments revealed concern regarding the use of nut butters, recommended in Nutrition Care Plan B and C, in early weaning foods, and allergic risk. However, recent studies suggest that although there are insufficient data to demonstrate that early introduction of peanut into infants’ diets – between 4 and 6 months of age – would reduce risk of developing a peanut allergy,^76^ early introduction of peanuts is not considered unsafe^77^ and as nut butters are a nutrient-dense food source the recommendation to fortify complementary foods with them has been included within the pathway as there is an extensive body of research considering their use in the form of Ready-to-Use Therapeutic Foods.^78^

Other work from our centre suggests that a nutritional pathway can be readily and accurately implemented in a healthcare setting improving nutritional care, growth, and clinical outcomes in vulnerable patient populations.^79^ ^,^ ^80^ The next stage of this quality improvement work is to implement the described nutritional pathway within a feasibility study. Part of this will include consideration of whether monitoring nutrition intake and growth using a digital home monitoring program is easy, feasible, and acceptable for parents and healthcare professionals (Fig 3; Supplementary material 3). We will use qualitative and quantitative methods^80^ to define the outcomes needed for a larger multicentre study to evaluate whether this approach does actually improve growth among infants with CHD before surgery.

There are a number of limitations to this work, the principal one being that consensus processes have inherent bias and a heavy reliance on the opinions of experts. There is also no standardised methodology for completing modified Delphi or Delphi processes and as such the recommended sample size and required response rate varies. The challenge with having a small group of experts within one field is that their opinions may show little variability, limiting the range of options considered in achieving consensus. A larger group of experts are likely to deliver a broader range of expertise, in turn making it more challenging to achieve consensus.^81^ Paediatric dietitians are usually the key healthcare professionals involved in the nutritional care of infants with CHD, and thus using their nutritional expertise for this modified Delphi process was appropriate. A total of 52 dietitians provided some input whether as part of the BDA meetings, online survey, or regional stakeholder meeting. This suggests there was high stakeholder engagement with the contents of the nutritional pathway and the need to standardise nutritional practices for this vulnerable cohort. They had a range of experience of Regional and Tertiary level 3 Cardiac centres, which ensured that the views of a wide range of opinions was taken into account. As the literature used for the development of the nutritional pathway was based on international research and practice, it is anticipated that the principles presented within the pathway are transferable to other healthcare systems.

## Conclusion

We have developed the first comprehensive, consensus-based Nutrition Pathway to guide nutritional support for infants with CHD before surgery and optimise growth in these vulnerable patients. Consensus regarding the format and content of the guideline was achieved among healthcare professionals working at specialist paediatric cardiac centres and at local district hospitals. We intend to implement the nutritional pathway in a feasibility study to determine whether it is practical to use and whether the pathway better supports growth in infants with CHD before surgery.

## Supplementary material

For supplementary material accompanying this paper visit http://dx.doi.org/10.1017/S1047951118000549. click here to view supplementary material
