# Supplementary material for: The development of a consensus-based nutritional pathway for infants with CHD before surgery using a modified Delphi process
Source: Cardiol Young. 2018 Apr 29;28(7):938–48. doi: 10.1017/S1047951118000549 (PMC5977758; doi:10.1017/S1047951118000549)
Supplement: Supplementary file 1 [file S1047951118000549sup.zip › S1047951118000549sup001/S1047951118000549sup001.docx]

| **uthors**  **Supplementary file Table 1: Nutrition in infants with CHD – review of recent evidence** | **Title** | **Year** | **Document Type** | **Level of evidence** | **Source origin (country of origin)** | **Aims/purpose of paper** | **Study population** | **Inclusion** | **Exclusion** | **Sample size** | **Methodology** | **Intervention type** | **Results** | **Key findings relevant to scoping review** |
| --- | --- | --- | --- | --- | --- | --- | --- | --- | --- | --- | --- | --- | --- | --- |
| Anderson J.B. ([82](#_ENREF_82)) | Poor post-operative growth in infants with two-ventricle physiology | 2011 | Review | 3b | USA | The aim was to describe perioperative growth in infants undergoing surgical repair of two-ventricle CHD and assess for predictors of their pattern of growth | Infants with 2 ventricle physiology | Infants with 2 ventricle physiology CHD | Infants with facial, gastrointestinal, or neurologic anomalies, trisomy chromosomal abnormality, birth weight < 2500 grams, or those transferred to another institution before discharge home were excluded | 76 | Full-term infants who had surgical repair of two-ventricle CHD enrolled in a retrospective cohort study following a larger prospective study | None | Median; age at surgery was 5 days (range 1 to 44), WAZ at surgery was -0.2 (-2.9 to 2.8); at discharge WAZ -1.2 (-3.4 to 1.8); change in WAZ from surgery to discharge was -1.0 (range -2.3 to 0.2). Delayed post-operative nutrition (p < 0.001)& reintubation following initial post-operative extubation (p = 0.001) were assoc with < WAZ | Infants undergoing repair of two-ventricle CHD had poor growth in the post-operative period which may be ameliorated by early initiation of post-operative nutrition |
| Benzecry S.G. ([83](#_ENREF_83)) | Interdisciplinary approach improves nutritional status of children with heart diseases | 2008 | Article | 4 | Brazil | To investigate the role of an interdisciplinary team in improving the nutrient intake and nutritional status of children with heart diseases | Children with CHD | Children with CHD | None | 35 | Prospective longitudinal case series | None | At the start prevalence of malnutrition 57%. Stunting and wasting were more frequent among patients with pulmonary hypertension and/or uncompensated congestive heart failure. Follow-up analyses showed > WHZ (-1.17 ± 1.03 vs. -0.32 ± 1.08, p<0.01); HAZ (-1.09 ± 0.96 vs. -0.51 ± 1.36, p<0.01). In patients with pulmonary hypertension or uncompensated congestive heart failure, the height-for-age index remained unchanged. Energy intake did not differ (112.0 ± 20.4 and 119.0 ± 18.0 kcal/kg/day) but significant > in intakes of micronutrients over the study period | Intervention by an interdisciplinary team improved the nutrient intake & nutritional status of patients overall. This however, was still insufficient to improve growth in the subgroup with pulmonary hypertension or uncompensated congestive heart failure. Nutritional support should be made routine in the treatment of children with heart disease. |
| Blasquez A. ([46](#_ENREF_46)) | Evaluation of nutritional status and support in children with congenital heart disease | 2016 | Article | 4 | France | To determine the prevalence of malnutrition in children with congenital heart disease | Infants < 6 months of age with CHD | Infants < 6 months of age with CHD | Infants > 6 months of age | 125 children four groups: no pulmonary hypertension (PH) or cyanosis (group 1, n=47), isolated cyanosis (group 2, n=52), isolated PH (group 3, n=16), and PH and cyanosis (group 4, n=10) | Retrospective chart review; prevalence of moderate to severe malnutrition (weight/weight for height <80%), caloric intake and medications were compared between the four groups | None | Moderate malnutrition was more common group 4 (100%) vs. (group 1, 20%; group 2, 16.7%, group 3, 50%; P<0.05). Low oral caloric intake was more common in group 3 (71.4%) & group 4 (75%) vs. group 1 (28%) and 2 (28.6%) (P<0.05). Food enrichment was practised 50% of group 4 but was not common in other groups (group 1, 15.8%; group 2, 8.6% & group 3, 11.1%; P<0.05). Enteral feeding was more common in groups 3 (33.3%) & 4 (50%) than in groups 1 (15.8%) or 2 (14.3%; P<0.05) | Moderate or severe malnutrition is present in 15% of children with CHD, and it is more frequent in case of PH. Half of these children demonstrate low caloric intake, whereas few have proper nutritional support |
| Boctor D.L.  ([68](#_ENREF_68)) | Nutrition after cardiac surgery for infants with congenital heart disease | 1999 | Article | 4 | Canada | To determine postoperative nutrition support patterns & identify factors associated with rate of weight gain in infants <1 year | Children with CHD | Children with CHD, gastrostomy insertion | None | None | Retrospective chart review | None | Median; age at surgery 2.7 months (range, 1 day to 9 months), daily weight change during the postoperative ward stay was -11 g/d (range, -145 to +84 g/d), a net gain in 8 (36%), net loss in 14 (64%) . Rate of weight change at the time of hospital discharge was related to feeding practice: median weight gain; bottle fed 20 g/d (range, -100 to +73 g/d); breast fed supplemented with bottle feeds 5 g/d (range, -83 to +43 g/d); exclusively breast fed infants lost a median of 49 g/d (range, -80 to -23 g/d) (p = 0.04). Those who were meeting a >% energy requirements by the time of hospital discharge had higher rates of weight gain (r = 0.78; p =0 .003). | The authors found that rate of weight gain were not related to cardiac lesion or hospital length of stay. Weight gain after cardiac surgery in infants is sub-optimal is related to feeding practices. Greater attention to achieving energy requirements during postoperative recovery is necessary, especially in breast fed infants |
| Carpenter J.L ([67](#_ENREF_67)) | Feeding gastrostomy in children with complex heart disease: when is a fundoplication indicated? | 2016 | Article | 4 | USA | To review all CHD patients who underwent feeding gastrostomy placement from 1/1/2004 to 4/7/2015. | Patients with CHD who underwent feeding gastrostomy placement | All patients with CHD who underwent gastrostomy placement | None | 111 patients | Retrospective chart review; Demographic data, cardiac diagnoses, operative details, post-operative complications, and the need for GJ feeding and fundoplication | None | 111 patients had a gastrostomy. Median age surgery was 37 weeks (3 weeks–13.7 years); av. weight was 5.3 ± 4.9 kg. 3 had a major complication (need for return to theatre or peri-operative death <30 days).3 needed fundoplication. 56 patients (62 %) had gastrostomy feeds, of which 7 (13 %) patients require GJ feeds. | Children with CHD will tolerate gastrostomy placement well with minimal major complications. Results support selective use of fundoplication in those with CHD who require a feeding gastrostomy |
| Ciotti G. ([47](#_ENREF_47)) | Nutritional support via percutaneous endoscopic gastrostomy in children with cardiac disease experiencing difficulties with feeding | 2002 | Article | 4 | UK | To review the impact of gastrostomy placement to augment nutritional needs in children with CHD | Children with CHD | Children with CHD, gastrostomy insertion | None | None | Retrospective chart review | None | n=37 children CHD who underwent gastrostomy placement to improve nutritional needs. Patients were stratified into those with cyanotic heart disease, when saturations of oxygen were less than 95%; those with non-cyanotic heart disease with saturations greater than 95%, vs. minor cardiac disease associated with a systemic disorder. Each group was compared to control children matched for age, gender, and diagnosis. WAZ was monitored over 295 days. WAZ improved in all CHD group. | Gastrostomy tube feeds improved the calories intake required to support malnourished children with CHD |
| Costello C.L. ([11](#_ENREF_11)) | Growth Restriction in Infants and Young Children with Congenital Heart Disease | 2015 | Article | 3b | Australia | Study aim was to determine the prevalence of growth restriction in infants and young children with congenital heart disease (CHD) and investigate the relationship between poor growth, feeding difficulties, cardiac classification, and nutrition intervention on outcomes | Infants and young children with CHD aged 0-3 years | Infants and children with CHD | None | 78 | Prospective observational cohort study | None | Many children have growth restriction e.g. z-score ≤-2; weight/age z-score (n = 18, 23%), height/age z-score (n = 16, 21%), weight/height z-score (n = 12, 18%). Increased length of hospital stay associated with preadmission; faltering growth (P = .009) & tube feeding (P = .002), diagnosis of cyanotic CHD (P = .015), feeding difficulties (P = .015). | Growth faltering is a problem in children with CHD. Preadmission faltering growth and poorer growth parameters were associated with an increased length of hospital stay. Nutritional screening from diagnosis may detect growth faltering, improve access to early nutrition intervention, and improve patient outcomes. Poor growth is a significant problem in infants and young children with CHD. This article identified pre-surgical screening may detect early growth faltering and allow access to early nutrition support. |
| Hehir D.A. ([52](#_ENREF_52)) | Normal interstage growth after the Norwood operation associated with interstage home monitoring | 2012 | Article | 4 | USA | To evaluate growth patterns of patients enrolled in interstage home-monitoring program (HMP), and the effect of a multidisciplinary team approach to nutrition management | Infants with univentricular physiology in HMP | Infants with univentricular physiology | Those not included in HMP | 148 | Retrospective cohort review | None | Growth velocity during interstage period was 26 ± 8 g/day. WAZ < from birth to discharge after S1P (-0.4 ± 0.9 to -1.3 ± 0.9; p < 0.001) but then > in interstage period to the time of S2P (-0.9 ± 1; p < 0.001). Factors assoc. with > growth during this period were male gender, greater birth weight, full oral feeding at S1P discharge. After S1P, infants enrolled in an HMP experienced normal growth velocity during the interstage period | Daily observation of oxygen saturation, weight change, and enteral intake together with implementation of a multidisciplinary feeding protocol was associated with good interstage growth & survival |
| Hofner G. ([69](#_ENREF_69)) | Enteral nutritional support by percutaneous endoscopic gastrostomy in children with congenital heart disease | 2000 | Article | 4 | Germany | To review the impact of gastrostomy placement to augment nutritional needs in children with CHD | Children with CHD | Children with CHD, gastrostomy insertion | None | None | Retrospective chart review | None | 4 / 8 children were followed for at least 6 months the age-matched body weight > 1 z score. 2 other patients > 0.5 z scores. In 7 children the tube was removed after 2.5 to 42 months since enteral support was no longer necessary. | The author’s report that apart from the initial reservations parental acceptance of PEG was good. They felt PEG is a safe and reliable technique to support enteral nutrition in children with severe CHD. |
| Irving SY ([44](#_ENREF_44)) | Resting energy expenditure at 3 months of age following neonatal surgery for congenital heart disease | 2013 | Article | 3b | USA | To determine the resting energy expenditure (REE), body composition, and growth in infants with CHD at 3 months of age. 2nd aim was to identify predictors of REE as compared with healthy infants | Infants with CHD | Infants with CHD | None | 93 | Sub group in prospective study | None | 93 infants, 44 (47%) with CHD. Of the infants with CHD, 39% had single ventricle (SV) physiology. There was no difference in REE related to cardiac physiology between infants with CHD and healthy infants or between infants with SV and biventricular (BV) physiology. Anthropometric z-scores for weight (-1.1 ± 1.1, P < 0.001), length (-0.7 ± 1.1, P < 0.05), and head circumference (-0.6 ± 1.2, P < 0.001) were lower in infants with CHD at 3 months of age. The percentage of body fat (%FAT) in postoperative infants with SV (24% ± 6, P = 0.02) and BV (23% ± 5, P < 0.001) physiology were lower than in healthy infants (27% ± 5), with no difference in REE. | At 3 months of age, there was no difference in REE between postsurgical infants with CHD and healthy infants. Infants with CHD had lower growth z-scores and %FAT. These data demonstrate decreased %FAT contributed to growth failure in the infants with CHD. |
| Jadcherla S.R. ([70](#_ENREF_70)) | Feeding abilities in neonates with congenital heart disease: A retrospective study | 2009 | Article | 4 | USA | The objective was to characterize feeding milestones related to transition to oral feeding among infants with CHD, and to identify factors impacting on feeding abilities | Feeding progress was tracked in the first hospitalization using a retrospective chart review | Infants with CHD | None | 76 infants (29 acyanotic, 47 cyanotic CHD) | Retrospective case series | None | Birth weights were no different. Cyanotic CHD need 3 x longer mechanical ventilation days, opioids and inotropes vs. acyanotic group. In both groups, prolonged respiratory support was correlated with time to reach maximal gavage feeds and nipple feeds (both, R2=0.8). In cyanotic group, delayed start of gavage feeds was correlated with time to attain maximal gavage feeds and nipple feeds (R2=0.8). Use of cardiopulmonary bypass in cyanotic CHD delayed feeding milestones & increased length of stay which was not seen in the acyanotic group | Neonates with acyanotic & cyanotic CHD group had significant delays with (a) feeding readiness, (b) successful gastric feeding, (c) oromotor readiness and (d) successful oromotor skills. Co-morbid factors that may directly influence the delay in feeding milestones include the (a) duration of respiratory support and (b) use of cardiopulmonary bypass. Delays in achieving maximum gavage and maximum nippling may suggest foregut dysmotility and oropharyngeal dysphagia. |
| Marino LV ([6](#_ENREF_6)) | A cross sectional audit of the prevalence of stunting in children attending a regional cardiac service | 2015 | Article | 4 | UK | To characterise prevalence of malnutrition in infants and children with CHD | Infants and young children with CHD aged 0-3 years | Infants and children with CHD | None | 1781 | Prospective observational cohort study | None | During the study period n=1781 children’s anthropometrical measures were recorded (Table 1). The prevalence of stunting was 28.4%. In older groups fewer children were stunted and the number of overweight/ obesity was line with the national average at 10.2% (5-19year-olds). During this time n=169 children < 60 months of age had cardiac surgery. In children who had weight for age z <-2 z scores [N=132, 30.23; ± 42.69] at the time of surgery vs. normal weight for age [N=64 SEM 21.44; ±19.85] had a significantly longer length of hospital stay of 8.79 days (p=0.0056). | There is a high level of stunting amongst a regional cohort of children attending cardiology service. A low WAZ resulted in a significantly longer length of hospital stay, which appeared to be independent of the complexity of the surgical procedure. In order to improve service provision to this vulnerable population group, we aim to investigate the feasibility of implementing a nutrition care pathway for children with CHD using a growth bundles to better promote weight and length gain. |
| Medoff-Cooper B. ([3](#_ENREF_3)) | Nutrition and growth in congenital heart disease: A challenge in children | 2013 | Review | 5 | USA | Growth failure secondary to feeding problems after complex neonatal cardiac surgery is well documented, but not well understood. The purpose of this review is to describe feeding and growth pattern in children with congenital heart defects | Infants with univentricular physiology | None | Non | None | Review | None | Almost 50% with univentricular heart defects required nasogastric or gastrostomy tube at discharge from surgery. Feeding difficulties persist beyond infancy causing parental anxiety. Infants are 'stunted' with weight & height below normal. 25%of these infants meet the definition of 'failure to thrive' in the 1st year of life. Short stature is a problem for many children, impacting on neurodevelopmental outcomes. A structured nutritional program may positively impact on growth during the interstage period prior to the superior cavopulmonary connection | Improving nutritional intake is a priority of the National Pediatric Cardiology Quality Improvement Collaborative. This initiative has enabled the development of best practices with the potential to ameliorate poor growth in children with CHD |
| Mitting R. ([16](#_ENREF_16)) | Nutritional Status and Clinical Outcome in Post term Neonates Undergoing Surgery for Congenital Heart Disease | 2015 | Article | 3b | UK | Study aim was to examine preoperative nutritional status, measured by admission weight-for-age z score, was associated with postoperative clinical outcome | Neonates undergoing surgery for congenital heart disease | Neonates undergoing surgery for congenital heart disease | Those undergoing ductus arteriosus ligation alone and those with coexisting noncardiac morbidity | 248 | Retrospective case series | None | Median: age was 7 days (2-15 d), weight was 3.3 kg (2.91-3.6 kg), weight-for-age z score was-0.77 (-1.44 to 0.01), 11% had a WAZ <-2 | A low WAZ score was independent of RACHS-1 score. In multivariable regression analysis, WAZ at admission had strong correlation with the number of days free of respiratory support (invasive and non-invasive ventilation) at 28 days (p < 0.0001) and with all-cause mortality at 1 year (p = 0.001). |
| Nydegger A. ([71](#_ENREF_71)) | Energy metabolism in infants with congenital heart disease | 2006 | Review | 5 | Australia | Review energy expenditure | Infants with CHD | Infants with CHD | None | None | Review | None | Growth faltering is common in children with CHD and is multifactorial in nature. Malnutrition at the time of surgery is associated with clinical corrective cardiac surgery. Energy imbalance is a major contributing factor. However, the published literature is difficult to interpret as studies generally involve small patient numbers with a diverse range of types and severity of cardiac lesions and genetic and/or prenatal factors. The age and time of corrective surgery affects the potential for nutritional recovery. Although the immediate postoperative period is characterized by a hypermetabolic state, low total and resting energy expenditure are reported within 24 h of surgery. After 5 d, resting energy expenditure returns to preoperative levels. Significant improvements in weight and growth occur within months after corrective surgery. However, limited postoperative recovery in nutritional status and growth occurs in infants with a low birth weight, intellectual deficit, or residual malformation. | Authors recommend that further studies are required to inform corrective CHD surgery timing in order to maximize nutritional outcomes as well as identifying those infants who may benefit from aggressive nutrition support before surgery |
| Pye S. ([72](#_ENREF_72)) | Parent education after new-born congenital heart surgery | 2003 | Review | 5 | USA | The aim was to provide a step-by-step guide regarding the information needs of families preparing to take their infants home after cardiac surgery. | Neonates with CHD | None | None | None | Review | None | Parents need to learn about care of neonates with CHD including; nutritional support safe prescription of medications. Parents also need to know when to call regarding complications and emergency situations. Potential complications and when to call their health care provider. | Parents are interested in information about infant development, medical issues specific to their child, as well as strategies to support normal development. Parent information materials, Web resources, and a family discharge teaching tool should be considered as part of discharge. |
| Radman M. ([73](#_ENREF_73)) | The effect of preoperative nutritional status on postoperative outcomes in children undergoing surgery for congenital heart defects in San Francisco (UCSF) and Guatemala City (UNICAR) | 2014 | Article | 4 | USA | The objective of this study was to determine the association between preoperative nutritional status & postoperative outcomes in children with CHD following cardiac surgery | Children with CHD | Children with CHD | None | 71 | Prospective, 2-center cohort study | None | Duration of continuous inotropic infusion (median, 66 hours; IQR 72 hours), preoperative BNP levels (median, 30 pg/mL; IQR, 75 pg/mL) assoc. with a lower preoperative triceps skin-fold z score (P <.05). Longer duration of any continuous inotropic infusion and higher preoperative BNP levels were also associated with lower preoperative prealbumin (12.1 ± 0.5 mg/dL) and albumin (3.2 ± 0.1; P <.05) levels. | Lower total body fat mass and acute and chronic malnutrition were assoc. with worse clinical outcomes in children undergoing surgery for CHD at UCSF, a well resourced centre. There was an inverse correlation between total body fat mass and BNP levels. Time of inotropic support and BNP increases as measures of nutritional status decrease, which the authors felt supported the hypothesis that malnutrition was associated with < myocardial function |
| Sables-Baus S. ([74](#_ENREF_74)) | Oral feeding outcomes in neonates with congenital cardiac disease undergoing cardiac surgery | 2012 | Review | 4 | USA | To describe growth and feeding in infants requiring neonatal surgery for CHD | Neonates undergoing surgery for congenital heart disease | Neonates undergoing surgery for congenital heart disease | None | 56 | Retrospective cohort review | None | 23% infants had genetic syndromes, 45% required pre-operative mechanical ventilation. Median time from birth to surgery - 8.4 days, 29 infants fed orally before surgery. The mean time from surgery to first oral feed was 12 hours. Time from surgery to oral feeding, the amount taken with first feeding, and cross-clamp times were significant predictors of oral feeding success, whereas the presence of comorbidity - genetic abnormality - & longer ventilator dependency predicted failure. 50% of neonates required a feeding tube at discharge, no infant discharged was solely breastfed. Discharge with a feeding tube was associated with > weight gain. | Neonates with CHD have many barriers to successful oral feeding at the time of hospital discharge. Enteral feeding guidelines focus on physiological stabilisation and do not always address the developmental milestones necessary to support oral feeding. Future prospective studies are necessary to identify multimodal strategies to optimise early feeding |
| Slicker J ([59](#_ENREF_59)) | Perioperative Feeding Approaches in Single Ventricle Infants: A Survey of 46 Centers | 2016 | Article | 4 | USA | To understand feeding practice amongst centres in the National Pediatric Cardiology Quality Improvement Collaborative for single ventricle neonates | 56 paediatric cardiac surgical centres was conducted | Centres participating in the National Pediatric Cardiology Quality Improvement Collaborative | None | 56 centres | Web based survey questionnaire | None | 46 (82%) completed a survey. Feeding before surgery was common in single ventricle infants (30/46; 65%). Those who did not feed preoperatively were concerned about the risk of necrotizing enterocolitis (16/16; 100%), use of umbilical artery catheter (12/16; 75%), prostaglandin infusion (9/16; 56%). In the postoperative period, most centres used an “internal guideline” (21/46; 46%) or an “informal practice” (15/46; 33%) to determine feeding readiness. | Considerable variation exists in feeding practices for infants with single ventricle congenital heart disease among 46 centres participating in a quality improvement collaborative. Although most centres generally feed infants preoperatively, feeding practices remain centre-specific. Variability continues in the immediate post-operative and interstage periods. Further opportunities exist for investigation, standardization and development of best-practice feeding guidelines. Describes immediate preoperative surgical period. Web based survey - so unable to present guidelines or a summary of feed practices with regards to nutritional needs and supporting growth. |
| Tandberg B.S. ([75](#_ENREF_75)) | Feeding infants with CHD with breast milk: Norwegian mother and child cohort study | 2010 | Article | 4 | Norway | The aim was to explore the prevalence of breast milk feeding (BMF) of infants with CHD in first 6 months of life vs. general population | A subsample Norwegian Mother and Child Cohort Study conducted by Norwegian Institute of Public Health | 60 600 mothers completed a questionnaire about infant feeding at 6 months postpartum. Infants with moderate/severe CHD (n = 131) identified via nationwide CHD registry, in addition to n=65 infants with CHD co-morbidity | None | 196 | Feeding questionnaire | None | Child age 2-6 months, mothers of infants with CHD hazard ratio (HR) of 1.69 of weaning their child vs. controls. Mothers of infants with CHD with comorbidity weaned at an even faster rate (HR 3.54). At 6 months, 9.9% of infants with CHD predominately breastfed, 64.1% continued to receive some breast milk, only 26% were fed no breast milk. For infants with CHD with comorbidity, corresponding percentage of those who were predominately, mixed or not breastfed were 7.7%, 43.1% and 49.2%, respectively. | Although CHD alone & CHD with comorbidity > risk that mothers weaned breastfeeding earlier, a high percentage continued some breastfeeding. Future studies should investigate factors that support continued BMF even in the most severely affected children with CHD, in addition to the use of term breastmilk fortifiers |
| Toole B.J. ([14](#_ENREF_14)) | Perioperative nutritional support and malnutrition in infants and children with congenital heart disease | 2014 | Article | 4 | USA | To assess the effect of nutritional status and cardiovascular risk on hospital outcomes after CHD in infants and children | Young children following cardiac surgery | Children < 24 month, length of ICU stay > 48 hours | ICU stay < 48 hours, children > 24 months | 124 | Retrospective case series | None | RACHS-1 score (1-3) n=99, RACHS-1 scores of (4-6) n=30). Prevalence of acute malnutrition 51.2% & chronic malnutrition 40.5%. Median hospital stay for mild, moderate, and severe chronic malnutrition was 31, 10, and 22.5 days, respectively, vs. normal, 15 days (Kruskal-Wallis, P<.005). The average energy and protein requirements met on day 7 were 68±27 (SD) % and 68±40%, respectively. | Almost 50% of patients were malnourished at surgery; 66% patients met their kcal and protein requirements by day 7. To improve hospital outcomes, care should be taken to optimize the nutritional condition of infants/ children prior to and following cardiac surgery to improve hospital outcomes |
| Trabulsi JC  ([45](#_ENREF_45)) | Total energy expenditure of infants with congenital heart disease who have undergone surgical intervention | 2015 | Article | 3b | USA | An observational study of infants with CHD and surgical intervention within the first 30 days of life and healthy infants of similar age was completed | Infants with CHD | 3-month-old infants (n = 15 CHD, 12 healthy) and 12-month-old infants (n = 11 CHD, 12 healthy) | None | 40 | Prospective study | Deuterium doubly labelled water | TEE for CHD infants was not significantly different from healthy infants at 3 and 12 months; TEE in CHD infants was 36.4 kcal/day higher (95 % CI -46.3, 119.2; p = 0.37) and 31.7 kcal/day higher, (95 % CI -71.5, 134.8; p = 0.53) at 3 and 12 months, respectively, compared to healthy infants. | During the first year of life, TEE of infants with CHD and interventional surgery within the first month of life was not different than age-matched healthy infants. When calculating TEE of ≤12-month-old infants with CHD who have undergone surgical intervention |
| Tregay J ([4](#_ENREF_4)) | “I was so worried about every drop of milk” – feeding problems at home are a significant concern for parents after major heart surgery in infancy | 2017 | Article | 4 | UK | To understand parent experiences of caring for a child with complex needs after major CHD surgery | Parents of 20 children (aged <1–5 months at hospital discharge) | Parents of infants who had undergone open heart surgery and subsequently died or been readmitted unexpectedly to intensive care following their initial discharge home | None | 20 | **Qualitative interviews** | None | Caring for a child with CHD after surgery is demanding. Feeding difficulties is one of the most significant parent stressors. | Health professionals near to the parents can be a good source of support for parents, only if they understand the needs of CHD infants and have appropriate expectations of weight gain. Specialist surgical centres should try to address some of the issues of parental stress around feeding/weight before hospital discharge. Qualitative work exploring themes around feeding. |
| Uzark K. ([76](#_ENREF_76)) | Interstage feeding and weight gain in infants following the Norwood operation: Can we change the outcome? | 2012 | Article | 3b | USA | The purpose of this study was to examine the impact of feeding strategy on interstage weight gain | Infants with univentricular physiology in HMP | Infants with univentricular physiology | Those not included in HMP | 158 | Prospective cohort study | None | Discharge feeding regimens were; oral 52%, oral with enteral feed supplementation 33%, nasogastric/gastrostomy tube only 15%. Significant differences in av. daily interstage weight gain among feeding groups; oral only 25.0g/day, oral/tube 21.4 g/day, tube only 22.3g/day p = 0.019. Tube-only-fed infants were significantly older at Stage II (p = 0.004) & a significantly greater change in WAZ (p = 0.007). Overall weight gain rate was 16-32 g/day, similar to infant norms. The rate of weight gain < over time, with earlier decline observed for oral- and oral/tube-fed infants (<15 grams per day at 5.4 months) vs. enteral-only-fed infants (<15 grams per day at 8.6 months). | Following Stage I palliation, infants discharged on oral feeding gained weight better than those requiring NGT |
| Wong J.J.M. ([28](#_ENREF_28)) | Nutrition Support for Children Undergoing Congenital Heart Surgeries: A Narrative Review | 2015 | Article | 3b | USA | The review aimed to consider the various aspects of nutrition in critically ill children with CHD; (1) energy expenditure, (2) perioperative factors contributing to energy metabolism, (3) bedside practices which may allow nutrient delivery to be optimised, (4) medium- to long-term impact of energy balance on clinical outcomes | Infants with CHD | Infants with CHD | None | None | Narrative review | None | The development of a nutrition algorithm aiming to optimize nutrition of children in the perioperative period with the aim of improving nutritional status and surgical outcomes | Narrative review - of the development of a nutritional algorithm to be used during the peri-operative period, although does not guide pre-operative support in infants awaiting surgery. |
