# Supplementary material for: The development of a consensus-based nutritional pathway for infants with CHD before surgery using a modified Delphi process
Source: Cardiol Young. 2018 Apr 29;28(7):938–48. doi: 10.1017/S1047951118000549 (PMC5977758; doi:10.1017/S1047951118000549)
Supplement: Supplementary file 1 [file S1047951118000549sup.zip › S1047951118000549sup001/S1047951118000549sup002.pdf]

Delphi consensus - Nutrition Guidelines in infants with CHD

Thank you for visiting our questionnaire page.

Why are we doing this Dephi consensus?
Congenital heart disease (CHD) is one of the most common birth problems in infants, found in 9 out of every 1,000 babies born. We have shown poor growth in infants with CHD prior to surgery results in increased length of stay and post surgical morbidity.

The aim of these nutritional guidelines is to support the development of growth in infants (< 12 months age) with congenital heart disease (CHD) prior to surgery. The draft guidelines have been developed by the Paediatric Cardiology Dietetic/ SLT team at University Hospital Southampton NHS Foundation Trust (UHS), with support from the British Dietitian Association Paediatric Cardiac Interest Group. This questionnaire forms part of a Delphi consensus which we want to ask you as experts about.

What happens next?
As experts taking part in this Delphi study you will be asked whether you agree or disagree with a set of statements regarding nutrition support in infants with CHD. There will also be an opportunity to provide comments as to whether you feel there is anything missing from each section. The study is anonymous to make sure everyone has an equal say. This Delphi study will be completed in 2 rounds using an on-line survey, with a final face to face meeting involving the British Dietitian Association Paediatric Cardiac Interest Group at UHS to validate the guidelines.

To be involved, simply complete this online questionnaire. It should take no longer than 5 - 10 minutes. It is entirely optional. If you do not wish to take part simply close the questionnaire. However we are really grateful for your opinions. There are no specific benefits to you from taking part but no risks either. All information you provide will be anonymous and cannot be withdrawn once you submit it. By completing the questionnaire you agree to participate in this process.

Section 1. Step 1 - Assess & classify nutrition risk

The aim of these nutritional guidelines is to support the development of feeding skills and growth in infants (< 12 months age) with congenital heart disease (CHD) prior to surgery. There are five steps within the nutrition guidelines and these questions will go through each section in sequence.

Using the scale from strongly disagree to strongly agree, please indicate the extent to which you agree or disagree with the following statements or questions.

If you have any additional comments to add please use the comments box at the end of each section.

Step 1 - the nutritional needs of infants with CHD will depend on the type of cardiac lesion

Step 1: Assess & classify nutrition riskÂ

All levels of health care professionals would be able to use these guidelines?

Â

Strongly disagree	1	2	3	4	5	6	7	8	9	10	Strongly agree
	○	○	○	○	○	○	○	○	○	○	

The nutritional needs of infants with CHD will depend on the type of cardiac lesions?

Strongly disagree	1	2	3	4	5	6	7	8	9	10	Strongly agree
	○	○	○	○	○	○	○	○	○	○	

It is important to develop someÂ nutrition guidelines for infants with CHD to improve growth before surgery?

Strongly disagree	1	2	3	4	5	6	7	8	9	10	Strongly agree
	○	○	○	○	○	○	○	○	○	○	

Infants with these cardiac lesions will have a lower nutrition risk

Stongly disagree	1	2	3	4	5	6	7	8	9	10	Strongly agree
Patent ductus arteriosus â€“ (if early surgery)	○	○	○	○	○	○	○	○	○	○	
Atrial septal defect	○	○	○	○	○	○	○	○	○	○	
Cor triatriatum	○	○	○	○	○	○	○	○	○	○	
Total anomalous pulmonary venous drainage	○	○	○	○	○	○	○	○	○	○	
Pulmonary stenosis	○	○	○	○	○	○	○	○	○	○	

Please write theÂ names, if any, of CHD lesions you feel should be included in the lower nutrition risk listÂ

Infants with these cardiac lesions will have a higher nutrition risk

Stongly disagree	1	2	3	4	5	6	7	8	9	10	Strongly agree
Coarctation of aorta	○	○	○	○	○	○	○	○	○	○	
Pulmonary atresia	○	○	○	○	○	○	○	○	○	○	
Tetralogy of Fallot	○	○	○	○	○	○	○	○	○	○	
Atrial septal defect â€“ (severe lesion)	○	○	○	○	○	○	○	○	○	○	
Transposition of great arteries	○	○	○	○	○	○	○	○	○	○	
Ventricular septal defect â€“ (moderate to large)	○	○	○	○	○	○	○	○	○	○	
Arterioventricular septal defect	○	○	○	○	○	○	○	○	○	○	
Hypoplastic left heart syndrome	○	○	○	○	○	○	○	○	○	○	
Truncus arteriosus	○	○	○	○	○	○	○	○	○	○	
Aorto pulmonary window	○	○	○	○	○	○	○	○	○	○	
Patent ductus arteriosus (large/delayed surgery)	○	○	○	○	○	○	○	○	○	○	
Tricuspid atresia	○	○	○	○	○	○	○	○	○	○	
Ebstein Anomaly	○	○	○	○	○	○	○	○	○	○	
Double outlet right ventricle	○	○	○	○	○	○	○	○	○	○	
Partial anomalous pulmonary venous drainage	○	○	○	○	○	○	○	○	○	○	

Please write theÂ names, if any, of CHD lesions you feel should beÂ included in the higher nutrition risk listÂ

Infants with more than 1 cardiac lesion AND a congenital or chromosomal abnormality will have a higher nutrition risk

Strongly disagree	1	2	3	4	5	6	7	8	9	10	Strongly agree
T21/18/13	○	○	○	○	○	○	○	○	○	○	
VACTRL/ CHARGE	○	○	○	○	○	○	○	○	○	○	
Gastrointestinal atresia	○	○	○	○	○	○	○	○	○	○	
Di-George Syndrome	○	○	○	○	○	○	○	○	○	○	
Congenital chylothorax	○	○	○	○	○	○	○	○	○	○	

Please write theÂ names, if any, of congenital abnormalities andÂ CHD lesions you feel should be included in the higher nutrition risk list

A cardiac lesion can be used to classify nutrition risk?

Strongly disagree	1	2	3	4	5	6	7	8	9	10	Strongly agree
	○	○	○	○	○	○	○	○	○	○	

Comments

Section 2. Step 2 - Assess & classify growth

Regular assessment of growth in an infant with CHD identifies early growth failure providing the opportunity to intervene

Regular assessment of growth in an infant with CHD identifies early growth failure providing an opportunity to intervene?

Strongly disagree	1	2	3	4	5	6	7	8	9	10	Strongly agree
	○	○	○	○	○	○	○	○	○	○	

An infant with lower nutrition risk will be

Strongly disagree	1	2	3	4	5	6	7	8	9	10	Strongly agree
Gaining an adequate amount of weight e.g. 10 g/kg/day	○	○	○	○	○	○	○	○	○	○	
Weight not more than 2 centiles below birth centile after 3 weeks of age	○	○	○	○	○	○	○	○	○	○	

Following a growth curve	○	○	○	○	○	○	○	○	○	○

An infant with higher nutrition risk will be

Strongly disagree	1	2	3	4	5	6	7	8	9	10	Strongly agree
Not gaining adequate amounts of weight e.g.	○	○	○	○	○	○	○	○	○	○	
Sustained weight drop of 2 centiles or more from birth after 3 weeks of age	○	○	○	○	○	○	○	○	○	○	
Flattening of growth curve	○	○	○	○	○	○	○	○	○	○	
Growth curve dropping downwards or losing weight	○	○	○	○	○	○	○	○	○	○	

Comments

Section 3. Section 3 - Eating & drinking

Prevent oral aversion by referring and involving a SLT early to assess feeding skills

To prevent oral aversion aÂ speech and language therapist (SLT) should reviewÂ those with high nutrition riskÂ

Strongly disagree	1	2	3	4	5	6	7	8	9	10	Strongly agree
	○	○	○	○	○	○	○	○	○	○	

AnÂ infant should have a SLT review if they

	Strongly disagree	1	2	3	4	5	6	7	8	9	10	Strongly agree
Shows signs of distress during or after a feed		○	○	○	○	○	○	○	○	○	○	
Breathing sounds are noisy/ wet during/after a feed		○	○	○	○	○	○	○	○	○	○	
Coughing, gagging or choking episodes		○	○	○	○	○	○	○	○	○	○	
Losing fluid from the mouth or fluid/ food remaining in the mouth		○	○	○	○	○	○	○	○	○	○	
Changes in breathing/ saturation levels during a feed		○	○	○	○	○	○	○	○	○	○	
An infant changes colour during or after a feed		○	○	○	○	○	○	○	○	○	○	
Regression of oral feeding skills or oro-motor difficulties		○	○	○	○	○	○	○	○	○	○	
Difficulty in moving from enteral feeds to oral intake		○	○	○	○	○	○	○	○	○	○	
Breath holding during a feed		○	○	○	○	○	○	○	○	○	○	

Comments

Section 4. Step 4 - What & how much is eaten/ drunk?

Restricted feed or food intake may impact on the infant's ability to maintain adequate nutritional status

Infants with a lower nutrition risk will ....

Strongly disagree	1	2	3	4	5	6	7	8	9	10	Strongly agree
Not vomit	○	○	○	○	○	○	○	○	○	○	
Drink 150ml/kg or above	○	○	○	○	○	○	○	○	○	○	
Keen to drink	○	○	○	○	○	○	○	○	○	○	
Finishes expected amount of infant feed	○	○	○	○	○	○	○	○	○	○	
Breastfeeds for expected duration	○	○	○	○	○	○	○	○	○	○	

Infants with higher nutrition risk will.....

Strongly disagree	1	2	3	4	5	6	7	8	9	10	Strongly agree
Vomit with most feeds	○	○	○	○	○	○	○	○	○	○	
Be fluid restricted or drink	○	○	○	○	○	○	○	○	○	○	
Only drinks a portion of the feed offered	○	○	○	○	○	○	○	○	○	○	
Require a nasogastric tube	○	○	○	○	○	○	○	○	○	○	

Comments

Section 5. Step 5 - Nutrition Care plans - A, B or C

Within this section of the guidelines there are 3 Nutrition Care plans; A, B or C depending on nutrition risk

Nutrition care plan A - Infants in this group will be

Strongly disagree	1	2	3	4	5	6	7	8	9	10	Strongly agree
Growing well	○	○	○	○	○	○	○	○	○	○	
Be keen to drink	○	○	○	○	○	○	○	○	○	○	
A CHD lesion with a lower nutritional risk	○	○	○	○	○	○	○	○	○	○	
Will require between 90 - 100 kcal/kg	○	○	○	○	○	○	○	○	○	○	
Require 1.5 g/kg protein (e.g. 2g protein per 150ml)	○	○	○	○	○	○	○	○	○	○	
Should have breastmilk or standard infant formula	○	○	○	○	○	○	○	○	○	○	
Weaning foods from 17 â€“ 26 wks age	○	○	○	○	○	○	○	○	○	○	
Should be reviewed by local team	○	○	○	○	○	○	○	○	○	○	

Comments

Nutrition care plan B - Infants in this group will be

Strongly disagree	1	2	3	4	5	6	7	8	9	10	Strongly agree
Not growing well	○	○	○	○	○	○	○	○	○	○	
Do not always finish feeds offered	○	○	○	○	○	○	○	○	○	○	
CHD lesion with a higher nutritional risk	○	○	○	○	○	○	○	○	○	○	
Shows signs of distress during feeds	○	○	○	○	○	○	○	○	○	○	
Fluid intake	○	○	○	○	○	○	○	○	○	○	
Will require between 110 - 120kcal/kg	○	○	○	○	○	○	○	○	○	○	

Will require 2.5g/kg protein										
Should have breastmilk/ infant formula & 30 - 50% energy/nutrient dense formula										
1 tsp nut butter in weaning foods from 17 - 26 wks of age										
Dietetic/ growth review every 2 weeks										

Comments

Nutrition care planÂ C - InfantsÂ in this group will be

Strongly disagree	1	2	3	4	5	6	7	8	9	10	Strongly agree
Losing weight/not growing well											
CHD lesion with higher nutrition risk											
Takes a long time to feed or tires easily											
Has difficulty feeding											
Fluid restricted											
Will require 120 - 150kcal/kg											
Will require up to 4g/kg protein											
Breastmilk/ infant formula & 50 - 80% energy/ nutrient dense feed											
1 - 2 tsp nut butter in weaning foods from 17 - 26 weeks											
Dietetic/ growth review every week											

Comments

Infants will no longer require intensive nutrition support when

Strongly disagree	1	2	3	4	5	6	7	8	9	10	Strongly agree
They have achieved catch up growth to 1 centile below birth weight	○	○	○	○	○	○	○	○	○	○	
They are 12 weeks post definitive surgery	○	○	○	○	○	○	○	○	○	○	

Comments

Section 6. Participant characteristics

Please check the button that best corresponds to you

What is your gender?

- ○ Male
- ○ Female
- ○ Prefer not to say

About you

	<30 years	30 - 39 years	40 - 49 years	50 - 59 years	> 60 years
How old are you?	○	○	○	○	○

What is your professional background?

Specialist Paediatric Nurse	Registrar	SHO	Consultant Cardiology	Consultant other	SLT	Dietitian	OT	Physio	Health visitor	Community nurse	Other
○	○	○	○	○	○	○	○	○	○	○	○

How long have you been working with infants with CHD?

< 2 years	2 - 4 years	5 - 6 years	7 - 8 years	9 - 10 years	> 10 years
○	○	○	○	○	○

How would you describe your place of work?

Paediatric Surgical Cardiac centre	Regional Hospital	District General Hospital	Community
○	○	○	○

Please provide an email address to allow us to send your individual summary

Thank you for taking time to complete this questionnaire.
