# Supplementary material for: The development of a consensus-based nutritional pathway for infants with CHD before surgery using a modified Delphi process
Source: Cardiol Young. 2018 Apr 29;28(7):938–48. doi: 10.1017/S1047951118000549 (PMC5977758; doi:10.1017/S1047951118000549)
Supplement: Supplementary file 1 [file S1047951118000549sup.zip › S1047951118000549sup001/S1047951118000549sup003.pdf]

Supplementary File 3:

Nutritional pathway for Infants with congenital heart disease before surgery

Using Steps 1 – 5 assess nutrition risk, classify growth, consider how and what an infant is drinking and eating to determine which nutrition care plan is appropriate e.g. A, B or C.

PATIENT ASSESSMENT SHEET: NUTRITION & FEEDING SKILLS			
**1. Assess CHD nutrition risk**	**2. Assess and classify infant's growth**	**3. How does the infant eat and drink?**	**4. What does the infant eat and drink?**

5. Chose a Nutrition care plan

A

B

C

6. Exit criteria

Dietetics

SLT

STEP 1 Assess and classify the infant with CHD nutritional needs

Key message 1
The nutritional needs of infants with CHD will depend on the type of cardiac lesion

Lower nutrition risk*	Higher nutrition risk*
• Patent ductus arteriosus – (if early surgery)<br>• Atrial septal defect<br>• Cor triatriatum<br>• Total anomalous pulmonary venous drainage<br>• Pulmonary stenosis<br>• Transposition of great arteries<br>• Coarctation of aorta<br><br>*This is not an exhaustive list and does not replace clinical judgement with respect to nutrition risk	• Pulmonary atresia<br>• Prostin dependent lesion<br>• Tetralogy of Fallot<br>• Atrial septal defect – (severe lesion)<br>• Ventricular septal defect – (moderate to large)<br>• Arterioventricular septal defect<br>• Hypoplastic left heart syndrome<br>• Truncus arteriosus<br>• Aortopulmonary window<br>• Patent ductus arteriosus (if large or delayed surgery)<br>• Tricuspid atresia<br>• Ebstein Anomaly<br>• Double outlet right ventricle<br>• Partial anomalous pulmonary venous drainage
Notes:<br>• Nutrition risk will be higher in infants with more than 1 cardiac lesion congenital or chromosomal abnormality such as:T21/18/13 /MVACTRL/ CHARGE/ Gastrointestinal atresia/ Congenital chylothorax/ Severe cardiomyopathy/ Syndromes: Noonan / Turners / Williams/ Di-George<br>• Premature infants or those with intra uterine growth retardation / absent or reversed end diastolic flow	

STEP 2 ASSESS AND CLASSIFY THE INFANTS GROWTH

Key message 2
Regularly plotting weight, length and head circumference on an appropriate growth chart in in infant s with CHD provides the opportunity to intervene where there are signs of growth faltering.

Lower nutritional risk	Higher nutritional risk
▪ Gaining adequate amounts of weight e.g. approx. 10g/kg/day and length ≈ 2cm per moth<br>▪ Weight/ length not more than 2 centiles below birth centile<br>▪ Following a growth curve which is not more than 2 centile s below birth centile	▪ Failure to gain adequate amounts of weight < 10g/kg /day and <2cm per month<br>▪ Sustained weight/ length drop of more 2 centiles or more from birth after 3 weeks of age<br>▪ Flattening growth curve or is dropping downwards e.g. losing weight

STEP 3 HOW DOES THE INFANT EAT OR DRINK?

Key message 3:
Prevent oral aversion by referring and involving a SLT early to assess feeding skills, particularly where there is a lack of progress or regression of feeding skills and associated clinical signs e.g. coughing, gagging or choking with feeds

STEP 4 WHAT AND HOW MUCH DOES THE INFANT EAT AND DRINK?

Key message 4:
Restricted feed or food intake may impact on the infant’s ability to maintain adequate nutritional status

Step 5 CHOOSING A NUTRITION CARE PLAN: A, B or C:
At each review using the flow diagram below decide the appropriate care plan outlined in the tables that follow

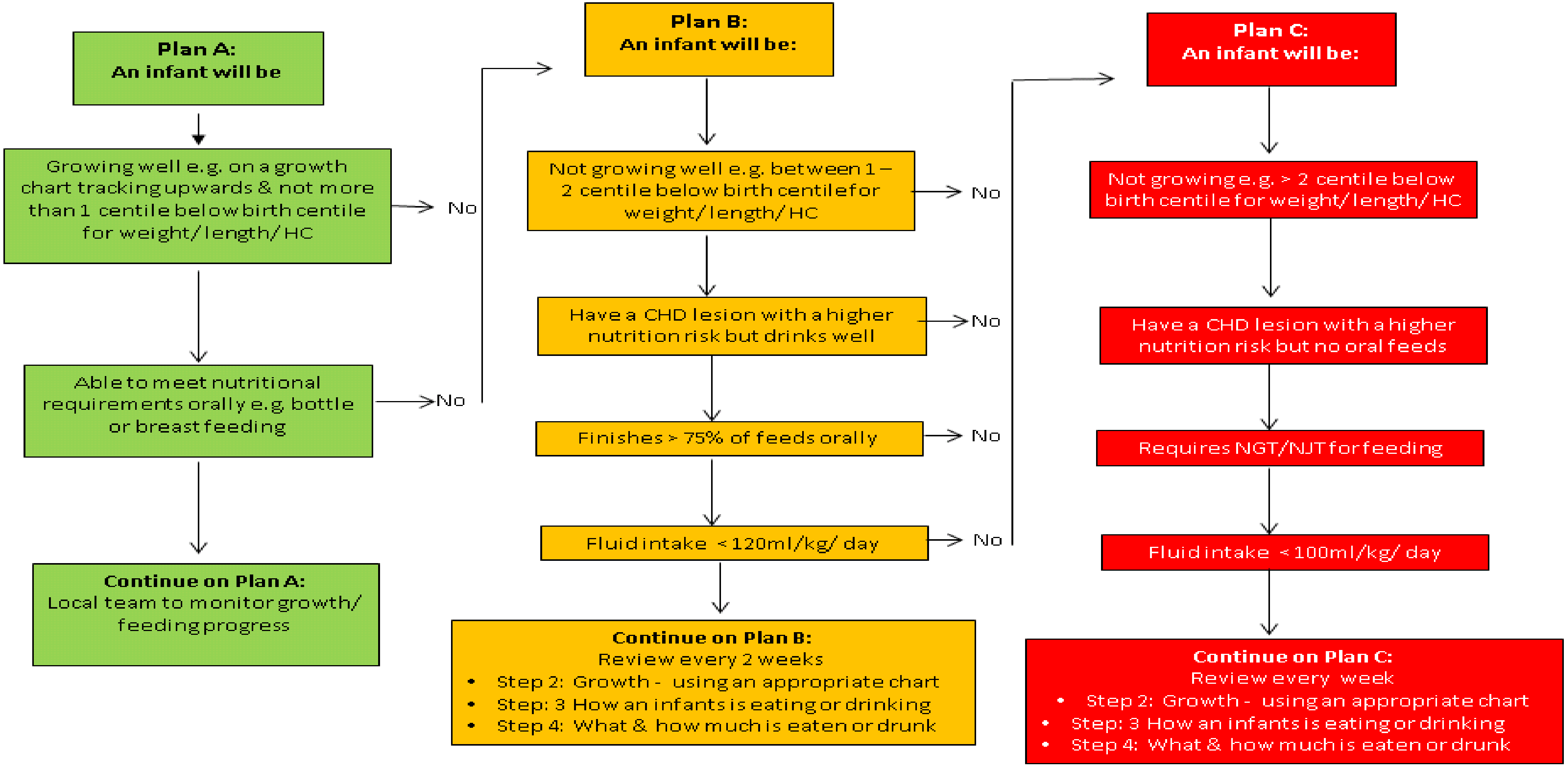

STEP 5 CHOOSE A NUTRITION CARE PLAN - A, B OR C

Key message 5: Involve a Paediatric Dietitian and Speech & Language Therapist in developing a nutritional and feeding care plan for an infant requiring plan B or C

Care Plan	Nutritional and feeding care plan
**A**	• Normal energy and protein requirements* 90 - 100kcal/kg protein 1.5/kg (e.g. 2g protein per 150ml)<br>• Normal fluid allowance e.g. 150ml/kg or above<br>• Breastfeeding or standard infant formula on demand<br>• Complementary food from 17 – 26 weeks if ready – 3 small meals/ day. Starches should form the basis of all meals, including veg/ fruit. Offer protein containing meals (chicken/ fish/ meat/ eggs/ pulses) at lunch / supper.<br>• Vitamins supplement to provide up to 10µg vitamin D<br>• Review by local team - refer to specialist centre with any concerns
**B**	• Approximately 10% extra energy* 100 – 110kcal/kg (protein contributing 9 -12% energy)<br>• Approximately 30 - 50% extra protein* (around 2.5g/kg protein)<br>• Breastmilk or standard infant formula in addition to 30 -80% of nutrition requirements from nutrient dense infant formula per day<br>• Complementary food from 17 – 26 weeks of age if ready – 3 small meals/ day. Starches should form the basis of all meals, including veg/ fruit. Offer protein containing meals (chicken/ fish/ meat/ eggs/ pulses) at lunch / supper. Around 6 months of age add ½ - 1 teaspoon of a nut butter or finely ground nuts to both main meals<br>• Vitamin supplement daily to provide up to 10µg vitamin D<br>• If there are any feeding issues refer to SLT<br>• Paediatric dietetic review growth in 2 weeks – if poor weight gain review earlier & move to plan C
**C**	• May be fluid restricted<br>• Approximately 10 - 20% extra energy* 120 - 150kcal/kg (protein contributing 10 -15% energy)<br>• Approximately 50 - 100% extra protein (up to 4g/kg protein – check renal function)<br>• Breastmilk or standard infant formula in addition to a minimum of 50 and up to 100% of nutrition requirements as energy/ nutrient dense infant formula or as overnight or nasogastric feeds<br>• Complementary food from 17 – 26 weeks of age if ready – 3 small meals/ day. Starches should form the basis of all meals, including veg/ fruit. Offer protein containing meals (chicken/ fish/ meat/ eggs/ pulses) at lunch/ supper. Around 6 months of age add 1 - 2 teaspoon of a nut butter or finely ground nuts all meals<br>• Vitamin supplement daily to provide up to 10µg vitamin D<br>• If there are any feeding issues refer to SLT<br>• Paediatric dietetic review of growth in 1 week

STEP 6: Exit criteria for dietetic & SLT support

**Dietetics**	• Post - operatively it may take 12 weeks or more for sufficient catch up growth to occur<br>• Nutrition rehabilitation will have been achieved when there is catch up growth to 1 centile below birth weight
**SLT**	• Eating and drinking skills are following appropriate stages for infant's presentation.<br>• Child is able to feed safely and independently and is growing appropriately.<br>• Feeding is an enjoyable experience for child and carer.<br>• All intervention advice and programmes are in place. Carers are skilled in carrying out recommended advice at which point infants should be discharged with support and re-referral options in place

*Based on actual weight rather than expected weight

Appendix C:
An infant should have a SLT review if they

- Shows signs of distress during or after a feed
- Breathing sounds are noisy/ wet during or after a feed
- Coughing, gagging or choking episodes
- Losing fluid from the mouth or fluid/ food remaining in the mouth
- There are changes in breathing rate / saturation levels of breath holding during a feed
- Or if an infant changes colour during or after a feed
- Regression of oral feeding skills or oro-motor difficulties
- Difficulty in moving from enteral feeds to oral intake
